# Use radiography rarely, not routinely, for hip hemiarthroplasty

**DOI:** 10.1007/s00068-021-01605-3

**Published:** 2021-01-22

**Authors:** Lucy Clare Maling, John Martin Lynch, Robert William Walker, Mark Ross Norton, Rory George Middleton

**Affiliations:** 1 Royal Cornwall Hospital, Royal Cornwall Hospitals NHS Trust, Truro, TR1 3LQ United Kingdom; 2 Present Address: Conquest Hospital, East Sussex Healthcare NHS Trust, Hastings, TN37 7RD United Kingdom

**Keywords:** Hip fracture, Hemiarthroplasty, Radiography

## Abstract

**Purpose:**

Hip hemiarthroplasty (HA) is a commonly performed operation. A post-operative radiograph forms part of the routine hip fracture pathway, although patients are often mobilised prior to this investigation. This study seeks to provide evidence for a pragmatic clinical change to optimise patient safety and allocate limited resources within the National Health Service (NHS).

**Methods:**

We undertook a retrospective database review of 1563 HA procedures to assess whether the routine ordering of check radiographs played an important role in a patient’s post-operative care.

**Results:**

18 (1.2%) mechanical complications led to a return to theatre within 6 weeks of the index procedure. All were dislocations. Ten had a normal post-operative radiograph and five had documented suspicion of dislocation prior to radiography. The post-operative check radiograph was the sole identifier of dislocation in only three patients (0.2%). All three of these patients were pre-morbidly bed bound and non-communicative due to cognitive impairment (AMTS 0/10).

**Conclusion:**

Unless a patient is pre-morbidly bed bound and cognitively impaired, routine post-operative radiography following HA surgery is of little clinical benefit, yet may carry considerable risk to the patient and cost to the NHS. A pragmatic compromise is to perform intra-operative fluoroscopic imaging.

## Introduction

Hip hemiarthroplasty (HA) is a commonly performed operation in elderly patients who have sustained a displaced intracapsular fracture of the neck of femur [1]. There are a number of risks associated with the operation, including dislocation, fracture, component malposition and retained acetabular cement [2]. These complications may be picked up on post-operative radiographs.

Many Trauma and Orthopaedic departments routinely obtain post-operative radiographs following HA prior to a patient’s discharge. In line with the National Institute for Health and Care Excellence (NICE) Guidance [1], patients who have undergone a HA are mobilised as soon after their operation as possible without waiting for radiographic clearance. This potentially obviates the usefulness of the radiograph in identifying complications that may be made worse with mobilisation.

Several studies have shown no benefit in routine radiography following hip fracture fixation [3–5]. However, its usefulness and validity have yet to be adequately investigated following HA surgery. In this study, we examine whether the routine ordering of check radiographs following HA surgery for hip fracture plays an important role in a patient’s post-operative care or clinical outcome.

## Methods

We undertook a retrospective search of our Hospital electronic database (Bluespier International, Droitwich, UK) and crossed referenced this with our Local National Hip fracture database. The capture period was 2012–2019 inclusive. Search words including “neck of femur fracture” “hemiarthroplasty”, “cemented hemiarthroplasty”, “uncemented hemiarthroplasty” and “bipolar hemiarthroplasty” were used to identify all HA operations undertaken for the neck of femur fracture. Data extracted included patient demographics, date of surgery, prosthesis used, fixation technique (cemented or uncemented), and further operation on the ipsilateral hip within a 6-week-period of the index surgery. We excluded revision HA and HA performed for any reason other than the neck of femur fracture.

Patients identified as having returned to the theatre had their paper and electronic records reviewed to understand the reason for their second operation. These were classified as either soft tissue (infection, haematoma or wound dehiscence) or mechanical (dislocation, fracture, component malposition or retained acetabular cement). These records were also reviewed for any documented clinical suspicion regarding a post-operative complication. In addition, post-operative check radiographs were reviewed to identify if any complications were evident on these images.

Patients who died within 6 weeks of their primary operation also had their images reviewed. Any patients who had not had a post-operative radiograph and had died within this time frame had their records reviewed to assess whether any suspicion of implant complication had been documented and also why they had not had a check radiograph.

A costing of obtaining a single pelvic radiograph was obtained from the accounts department of the hospital.

## Results

**Fig. 1:**
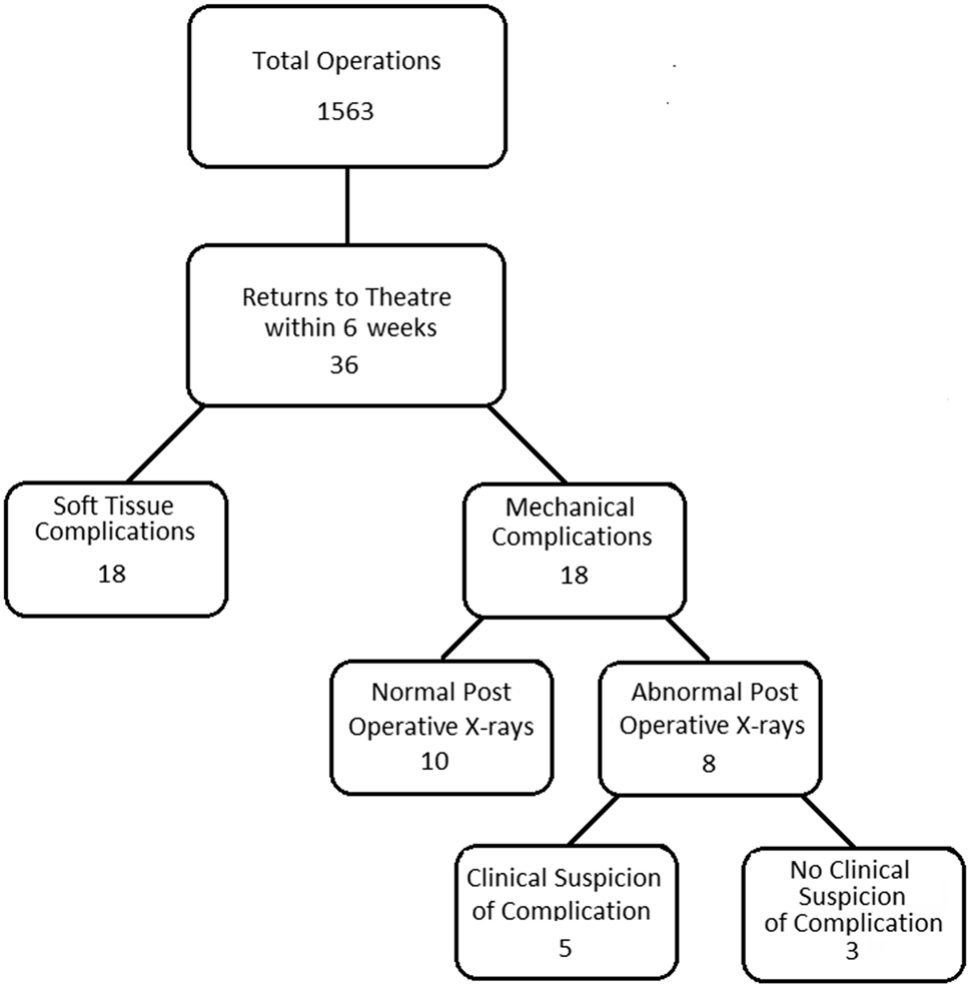
Breakdown of complication incidence and X-ray findings

1563 HA operations were identified, comprising 1522 cemented and 41 uncemented. Of the 1563 cases, 36 (2.3%) had complications which required a return to theatre within 6 weeks of their initial operation (Fig. 1). Complication rates were similar in cemented and uncemented groups. 18 (1.2%) patients were found to have had soft tissue complications. The remaining 18 (1.2%) had mechanical complications. All mechanical complications were dislocations; there were no cases of fracture, isolated component malposition or retained acetabular cement. 10 of the dislocations had normal immediate post-operative check radiographs.

The remaining eight patients had an identifiable mechanical complication on the immediate post-operative radiograph. Documented suspicion of a complication was noted prior to the patient having had their check radiograph in five of these eight cases. In the remaining three (0.2%), the check radiograph was the sole identifier of the complication. All three of these patients were immobile pre-morbidly and had documented diagnoses of advanced dementia with an Abbreviated Mental Test Score (AMTS) of 0/10 [6].

108 patients (6.5%) died within 6 weeks of having their operation. There were no complications picked up on the check radiographs of those that had them before they died. This left a total of 39 patients who had not had a check radiograph. No documentation of a possible complication with the prostheses was documented for any of these patients.

The cost of a single anterior–posterior view of the pelvis was calculated at £53. This was then multiplied by the number of check radiographs giving a figure of £80,772 spent over an 8-year period, equating to an excess of £10,000 per year.

## Discussion

This study indicates that if a patient is progressing well with their early rehabilitation, the likelihood that imaging will change the outcome of their care is small. More importantly, it also identifies a subset of at-risk patients where a post-operative radiograph is indicated. In this series, 0.2% had a complication which required a return to theatre identified by radiography alone. All of these patients were pre-morbidly bed-bound and poorly communicative due to severe cognitive impairment. In this group of patients, therefore, we would advise that a post-operative radiograph ought to be undertaken.

NICE recommends mobilising HA patients on the first post-operative day to prevent the complications associated with prolonged bed rest [1]. There is evidence to suggest that in other lower limb trauma, immediate post-operative radiographs rarely have an impact on clinical treatment [3, 7]. Only one previous study by Chakravarthy et al. [3] published in 2007, has examined radiographs for HA and found no complications in their subset of 460 patients. In addition, they established that nationally, 87% of surgeons surveyed would allow their patients to fully weight bear prior to check radiographs.

Post-operative radiographs provide details on the position and fixation of components. Surgeons may compare images to assess any changes later in the life of the prosthesis. This may be helpful when considering revision surgery, particularly for total hip arthroplasty (THA). However, elderly patients who have femoral neck fractures requiring HA have a reduced life expectancy [8]. As such, the position of the prosthesis is arguably of less concern as revision surgery for long term progressive implant failure is comparatively rare [9].

This study highlights the cost implications that routine radiographs have on an orthopaedic department. Approximately £10,000 a year was spent obtaining post-operative HA radiographs in this hospital. Phelps et al. [7] investigated the cost-effectiveness of routine post-operative radiographs following lower limb trauma surgery in the United States of America. They noted that, comparable to our findings, routine radiography altered the clinical course in only 0.3% of patients, at a cost of over $200,000,000 per annum. Other unseen costs are also incurred when obtaining post-operative radiographs, such as a potential delay to discharge [10].

The primary purpose of post-operative imaging is to identify mechanical complications as previously discussed. On this basis, our study suggests that, unless pre-morbidly bed-bound and with cognitive impairment, these are not necessary. However, we acknowledge the radiograph has other uses. It is also used to critically assess one’s performance during training or in the setting of complex surgery, novel implants or surgical techniques [11]. Radiographic evidence may also be of increasing importance in a medical culture that places emphasis on robust audit trails to satisfy governance and medico-legal accountability. With these arguments in mind, a pragmatic solution is to obtain a post-operative fluoroscopic image prior to the patient being transferred from the operating table.

Although this study was undertaken prior to the COVID-19 pandemic, its findings are paramount in this setting, where there is a greater drive than ever to streamline inpatient care and protect limited resources within the National Health Service (NHS) [12, 13]. The British Orthopaedic Association Standards for Trauma (BOAST), published in response to the coronavirus pandemic, support early mobilisation, judicious use of radiology services and expedited discharge [14]. The guidance also acknowledges that during this time, changes to standard practice may be required in the interest of clinical safety or resource protection.

A recent survey of 85 hospitals within the United Kingdom found that many had already implemented this change to their clinical practice, with the rate of post-operative HA radiography dropping from 98 to 74% during the COVID-19 pandemic [15]. Although numerous institutions have already introduced the proposed change in practice, this study is, to the authors’ knowledge, the first to identify those patients who may still require routine post-operative radiography.

We acknowledge the limitations of this study. Out of necessity, it was a retrospective review with no control group. It may be that the database review did not pick up all cases of hip HA surgery, though we are confident that the sample is large enough that it is representative of the population as a whole. Costing a check radiograph at £53 may be a largely arbitrary figure as it is a fixed overhead. However, we include it to help highlight other unseen costs such as the use of staff time and delay to discharge.

## Conclusion

Our results suggest that, unless a patient is pre-morbidly bed-bound and cognitively impaired, routine immediate post-operative radiography following HA surgery is of little clinical benefit, yet may carry a considerable cost to the NHS. A pragmatic compromise is to perform intra-operative fluoroscopic imaging.

## Data Availability

Data available on request via corresponding author.
